# Design and assembly of ternary Pt/Re/SnO_2_ NPs by controlling the zeta potential of individual Pt, Re, and SnO_2_ NPs

**DOI:** 10.1007/s11051-018-4244-0

**Published:** 2018-05-12

**Authors:** Elżbieta Drzymała, Grzegorz Gruzeł, Anna Pajor-Świerzy, Joanna Depciuch, Robert Socha, Andrzej Kowal, Piotr Warszyński, Magdalena Parlinska-Wojtan

**Affiliations:** 1 Institute of Nuclear Physics Polish Academy of Sciences, PL-31342 Krakow, Poland; 2 Jerzy Haber Institute of Catalysis and Surface Chemistry Polish Academy of Sciences, Niezapominajek 8, PL-30239 Krakow, Poland

**Keywords:** Catalytic nanoparticles, TEM characterization, Zeta potential, Pt, Re, SnO_2_ NPs, Nanocolloids

## Abstract

In this study Pt, Re, and SnO_2_ nanoparticles (NPs) were combined in a controlled manner into binary and ternary combinations for a possible application for ethanol oxidation. For this purpose, zeta potentials as a function of the pH of the individual NPs solutions were measured. In order to successfully combine the NPs into Pt/SnO_2_ and Re/SnO_2_ NPs, the solutions were mixed together at a pH guaranteeing opposite zeta potentials of the metal and oxide NPs. The individually synthesized NPs and their binary/ternary combinations were characterized by Fourier transform infrared spectroscopy (FTIR) and scanning transmission electron microscopy (STEM) combined with energy dispersive X-ray spectroscopy (EDS) analysis. FTIR and XPS spectroscopy showed that the individually synthesized Pt and Re NPs are metallic and the Sn component was oxidized to SnO_2_. STEM showed that all NPs are well crystallized and the sizes of the Pt, Re, and SnO_2_ NPs were 2.2, 1.0, and 3.4 nm, respectively. Moreover, EDS analysis confirmed the successful formation of binary Pt/SnO_2_ and Re/SnO_2_ NP, as well as ternary Pt/Re/SnO_2_ NP combinations. This study shows that by controlling the zeta potential of individual metal and oxide NPs, it is possible to assemble them into binary and ternary combinations.

**Electronic supplementary material:**

The online version of this article (10.1007/s11051-018-4244-0) contains supplementary material, which is available to authorized users.

**Graphical abstract:**
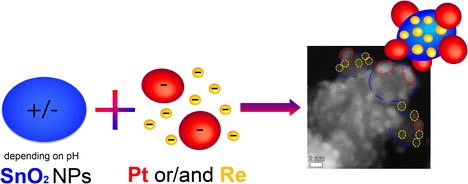
ᅟ

## Introduction

Nowadays, finding alternative sources of energy is an important global issue (dos Anjos et al. 2008; Li et al. 2013). The solution of this problem are ethanol fuel cells, which become more and more popular. This is related to the benefits of ethanol: it is less toxic compared to methanol, easier stored and transported than hydrogen, and available from renewable resources (Li et al. 2010; Du et al. 2011). However, the usage of ethanol as fuel generates various challenges, such as the difficulty to split the C–C bond and production of many by-products (Delpeuch et al. 2016). Therefore, the key challenge is to design and develop the appropriate type of catalysts. A good approach to obtain highly selective and active electrocatalysts for ethanol oxidation reaction (EOR), involves the preparation of multifunctional and multicomponent nanostructured particles (Antolini 2007; Antolini and Gonzalez 2011; Goel and Basu 2012; Zhang et al. 2017). These types of nanostructures are extensively synthesized and studied because they exhibit interesting properties, being key components in many catalytic applications. Very important is the possibility of precisely controlling the size, shape, and composition of the synthesized nanoparticles. Because catalysis is a surface effect, the catalyst needs to have the highest possible surface area, which is related to the size reduction (Sun et al. 2011). So far, the most promising group of nanocatalysts for ethanol oxidation are ternary catalysts based on mixtures of Pt, Rh, and SnO_2_ designed by the Adzic group (Kowal et al. 2009b; Li et al. 2010, 2013). This type of ternary nanocatalysts is also studied by other groups (Higuchi et al. 2014; Delpeuch et al. 2016). This is related to the unique and individual role of each component in the ethanol oxidation pathway. The role of Rh is to cleave the C–C bond in ethanol, while SnO_2_ provides OH species to oxidize intermediates and to free Pt and Rh sites for further ethanol oxidation (Li et al. 2013). Another important issue mentioned above, is the physical contact between the synthesized NPs. Kowal et al. (2009a) studied ethanol oxidation reaction (EOR) on ternary NP system containing Pt, Rh, and SnO_2_, showing a higher activity of such system, because the ethanol molecule has to be in contact with all the phases of the catalyst, which allows for a complete EOR. In their earlier work, they demonstrated the high efficiency of the catalyst of the same composition containing a Pt–Rh alloy (Kowal et al. 2009b). Another group (Higuchi et al. 2014) studied the Pt/Rh/SnO_2_ catalyst, but they prepared a nanocatalyst only with partial contact between the respective nanoparticles. The authors confirmed that it is not clear, if for EOR it is better to have a nanoalloy between the metallic elements or is a physical contact between them sufficient. The investigation by Park et al. (2008) shows that the control of alloy composition allows tuning the catalytic activity. On the other hand, Roth et al. (2005) indicate that alloy formation does not seem to be a crucial requirement for a superior electrocatalytic performance, as long as a close contact between both phases is achieved. Therefore, understanding the synergistic effect occurring between all three components of the designed catalyst, preparing standard samples consisting of these three nanoparticles, control of their size, composition and contact between them, are key steps in further development of this field. One of the strategies, which can lead to successfully interconnecting the desired nanoparticles, is based on controlled assembly of separately synthesized nanoparticles.

One of the main factors determining the interactions of nanoparticles in the suspension is their charge. The presence of this charge causes that, according to the DLVO theory, in the low-salt conditions, the nanoparticles repel each other and their suspension remains kinetically stable. However, in a mixture of negatively and positively charged nanoparticles, electrostatic attraction between them occurs resulting in heteroaggregation (or agglomeration). The experimentally accessible measure of the state of nanoparticles charge is their zeta potential. A high zeta potential (negative or positive), usually |*ζ*| > 30 mV, indicates the suspension stability. Generally, the charge of the metallic and metal oxide nanoparticles can be easily controlled by changing the pH of the suspension. The mechanism of surface charging is reported in detail in the monograph by Kosmulski (2009). However, it is well known that electrostatic interactions, besides pH, are strongly influenced by ionic strength and/or use of ligands for nanoparticle stabilization (Mori et al. 2009).

Aziz et al. (2013) analyzed the dependence of the zeta potential of aqueous suspension of SnO_2_ nanoparticles on the pH, prior to their electrophoretic deposition. They have observed negatively charged nanoparticles at pH values from about 4.5 to 11. On the other hand, Kosmulski (2009) measured the pH values of the isoelectric point for SnO_2_ particle suspensions from various sources, as ranging between 3.8 and 5.6, depending on the method of their synthesis.

Controlling the zeta potential of individual nanoparticles is especially important, when designing and connecting different NPs to form multi-metal nanoparticle structures. Selecting an optimal value of the zeta potential allows to connect different types of nanoparticles such as metals and oxides. For example, heterointerfaces of TiO_2_ and SnO_2_ NPs were formed by Siedl et al. (2012). By changing the pH of those nanoparticles, they have obtained oppositely charged suspensions, with zeta potential values of +24 and − 9 mV for TiO_2_ and SnO_2_ NPs, respectively, which allowed for the formation of a uniform network between them. The interaction of positively charged Pt-Ag alloy nanoislands with negatively charged graphene sheets resulted in the formation of hybrid composites for electro-oxidation of methanol (Feng et al. 2011).

The aim of the present study was to synthesize individually Pt, Re, and SnO_2_ nanoparticles and then, using the electrostatic interactions occurring between these nanoparticles, assemble them into binary Re/SnO_2_ or Pt/SnO_2_ and ternary Pt/Re/SnO_2_ NP combinations. To find the optimal condition for the heteroaggregation process to occur, the zeta potentials of the Pt, Re, and SnO_2_ nanoparticle solutions were determined depending on the synthesis method and suspension pH. The morphology and chemical composition of the Pt, Re, and SnO_2_ nanoparticles and their binary and ternary combinations were analyzed by transmission electron microscopy combined with EDS. The metallic and oxidation state was controlled by FTIR spectroscopy and X-ray photoelectron spectroscopy (XPS).

## Experiment

### Materials

All reagents were used as received. Tin (IV) chloride pentahydrate (SnCl_4_·5H_2_O), tin (II) chloride dihydrate (SnCl_2_·2H_2_O), hexachloroplatinic acid (H_2_PtCl_6_), ammonium perrhenate (NH_4_ReO_4_), and polyvinylpyrrolidone (PVP; average mol wt. 40.000) were purchased from Sigma-Aldrich. Other reagents such as citric acid (C_6_H_8_O_7_), ethylene glycol (C_2_H_6_O_2_), sodium borohydride (NaBH_4_), and ammonia solution (NH_3_·H_2_O) were acquired from Avantor. The SnCl_2_ and SnCl_4_ solutions were made by dissolving, respectively, SnCl_2_·2H_2_O and SnCl_4_·5H_2_O in distilled water or ethylene glycol (depending on the method).

#### Synthesis of SnO_2_ NPs

##### Citrate synthesis of SnO_2_(C)

A 0.1 M solution of tin (IV) chloride was prepared by dissolving 0.876 g of SnCl_4_·5H_2_O in 25 mL distilled water. Subsequently, 25 mL of 0.2 M citric acid solution was prepared and added to the above described tin chloride solution. The obtained solution was mixed at 50 °C for 30 min under magnetic stirring. In the next step, a 25% ammonia solution (NH_3_·H_2_O) was added drop-wise to the mixture under constant stirring. This was continued until the pH of the solution reached a value between 8 and 9. In this way, a white, cloudy solution was obtained, which precipitated. The latter one was washed with distilled water.

##### Polyol synthesis of SnO_2_(P) and microwave-assisted synthesis of SnO_2_(M)

The other two samples were synthesized according to the procedure described in detail in Drzymała et al. (2017). In the case of microwave-assisted and polyol syntheses, the heating time was extended to 16 min and 5 h, respectively. SnO_2_ nanoparticles obtained by the polyol method were precipitated, washed with ethanol, and dried. The resulting dry, slightly yellow powder was then suspended in a mixture of ethanol and water, sonicated, and used for binary connections and for further investigation.

#### Synthesis of rhenium NPs (colloidal method)

Based on the procedure proposed by Bedia et al. (2015), 5 mL of a 0.02 M solution of NH_4_ReO_4_ in distilled water was prepared. Additionally, different amounts of PVP were dissolved in 20 mL of distilled water. The amounts of PVP dissolved were adjusted in order to obtain PVP:Re molar ratio equal to 10. Next, both solutions were mixed and heated to 50 °C for 40 min under magnetic stirring. Additionally, argon gas was bubbled into the obtained mixture to remove the oxygen dissolved in the water, in order to avoid oxidizing conditions. Finally, 5 mL of a 0.5 M NaBH_4_ aqueous solution was introduced drop-wise under vigorous stirring to prepare a Re hydrosol. The Re hydrosol was stirred for 4 h after the addition of the reducing agent to allow complete reduction. The finally obtained solution was gray (the precipitate was black). Finally, the nanoparticles were washed with copious amounts of distilled water and ethanol. The pH of the obtained nanoparticle suspension after washing was about 5. As a control experiment, Re NPs were also synthesized in two other pH values, as shown in Fig. S1.

#### Synthesis of platinum NPs

The H_2_PtCl_6_ solution was prepared by dissolving 0.048 g H_2_PtCl_6_·6H_2_O in 40 mL of ethylene glycol. Next, the 0.5 M NaOH solution (diluted in EG) was used to raise its pH value to 12. The mixture was stirred to completely dissolve the precursor at ambient temperature, heated up to 160 °C, and maintained at this temperature for 3 h to ensure a complete reaction. During this reaction time, the initially orange solution turned into black at about 100 °C. The resulting nanoparticle suspension was precipitated and washed with ethanol and copious amount of distilled water. After washing, the pH of the obtained Pt NP solution was about 5. As a control experiment, Pt NPs were also synthesized in acidic pH values, as shown in Fig. S2.

#### Zeta potential measurements

The zeta potential distribution of all types of nanoparticles was determined by the microelectrophoretic method using Zetasizer Nano Series from Malvern Instruments. The Smoluchowski model was used in zeta potential measurements. Each value was obtained as an average of three subsequent runs of the instrument with at least 20 measurements. All experiments were performed in water at 25 °C.

#### Binary and ternary nanoparticle assembly method

First, the zeta potentials of the respective solutions containing Pt NPs, Re NPs, and SnO_2_ NPs were measured as a function of the pH. To produce binary Pt/SnO_2_ and Re/SnO_2_ NPs combinations, the pH of the respective nanoparticle solutions had to be adjusted, in order to have oppositely charged NPs. The charge values were obtained from the zeta potential measurements. After successfully creating the binary combination of Re/SnO_2_ nanoparticles, the Pt NP nanoparticle containing suspension was added. Prior adding Pt NPs, the zeta potential of binary combination (Re/SnO_2_) has been measured as a function of the pH. Pt was also washed several times with distilled water and ethanol until the pH has reached the acidic value of about 3–4. Both solutions were first sonicated and mixed separately. Subsequently, Pt nanoparticles were added drop-wise to the sonicated mixture of Re/SnO_2_. The resulting ternary mixture was sonicated together for some time and then stirred overnight.

#### TEM characterization

The morphology of the synthesized nanoparticles was examined by scanning transmission electron microscopy (STEM) using the high-angle annular dark-field detector (HAADF), in conventional and high-resolution mode. Selected area electron diffraction (SAED) patterns were acquired in the TEM mode. All these measurements were performed on a *C*_*s*_ aberration-corrected FEI Titan electron microscope operating at 300 kV equipped with a FEG cathode. Energy-dispersive X-ray spectroscopy (EDS) was used to analyze the chemical composition of the synthesized nanoparticles. The EDS mappings were performed on a Talos F200 FEI instrument operating at 200 kV equipped with a FEG cathode. The particle average size was evaluated based on the HRSTEM images taken from different areas of the TEM grids. For each sample, the diameter of 100 nanoparticles was measured.

#### FTIR spectroscopy

The Fourier transform infrared absorption (FTIR) spectra in the wave number range of 400–4000 cm^−1^ were acquired using an EXCALIBUR FTS-3000 spectrometer operating at room temperature and measured for the sample mixed with KBr. The 64 scans were averaged at a resolution of 4 cm^−1^. The sample was dried and sandwiched between two KRS-5 window disks. During the experiments, the spectrometer was purged with dry nitrogen. Baseline correction and normalization of FTIR spectra were performed.

#### XPS measurements

XPS characterization was carried out with an ESCA/XPS equipped with a semispherical analyzer EA15 (Prevac) using Al-Kα (1486.6 eV) radiation with a power of 180 W. The resolution of the spectrometer for the Ag 3d_5/2_ line was 1.0 eV, and the spectra were acquired at a pressure of 1 × 10^−9^ mbar.

## Results

### TEM characterization

**Fig. 1:**
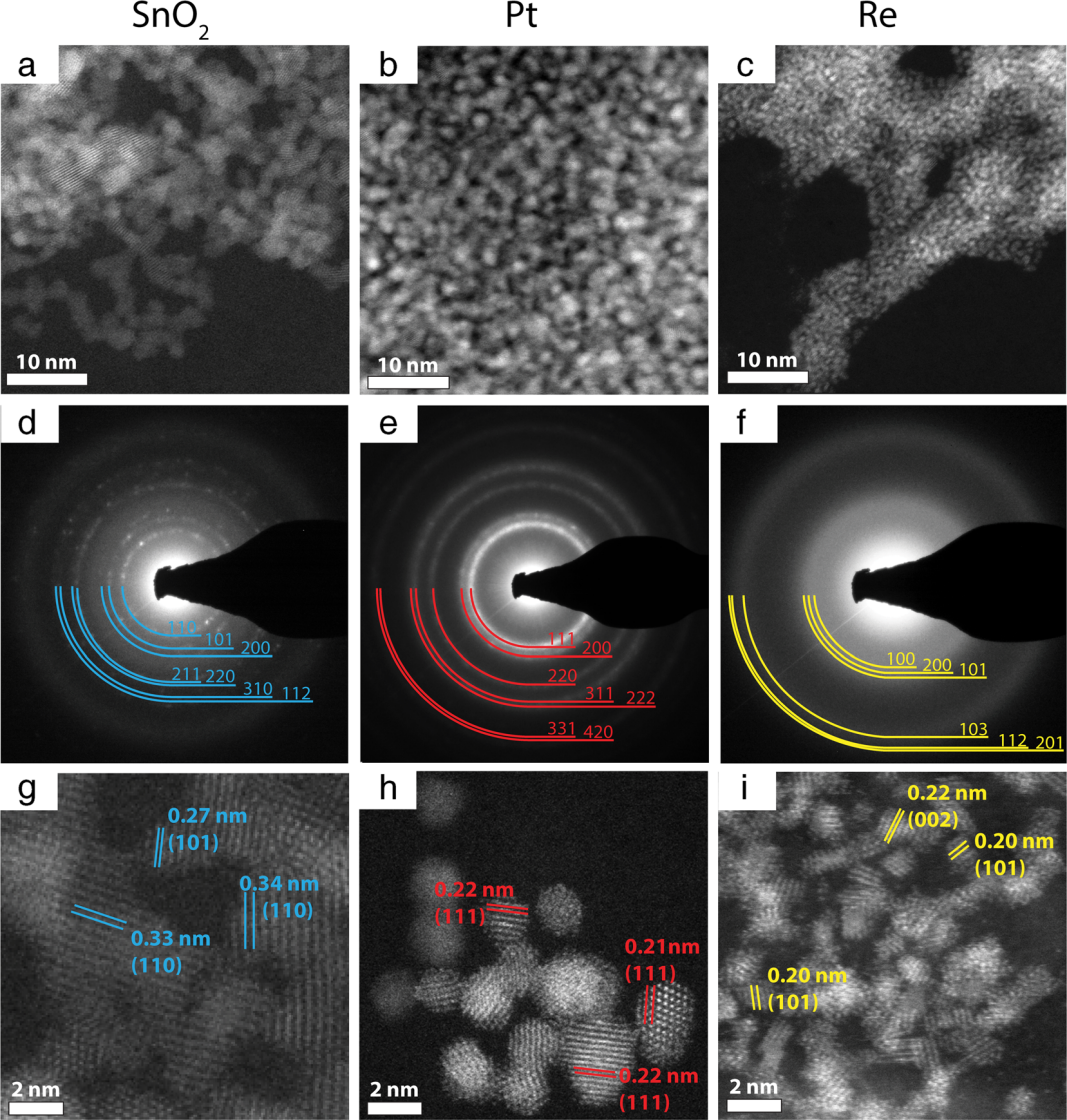
HAADF STEM overview (**a**–**c**), corresponding SAED patterns (**d**–**f**), and atomic resolution images (**g**–**i**) of SnO_2_ (*left column*), Pt (*middle column*), and Re NPs (*right column*)

The ternary particles were designed in such a way, so that the metal nanoparticles are supported by the tin oxide NPs. As a first step, the morphology of Pt, Re, and SnO_2_ nanoparticles was determined using the HRSTEM technique (Fig. 1).

**Fig. 2:**
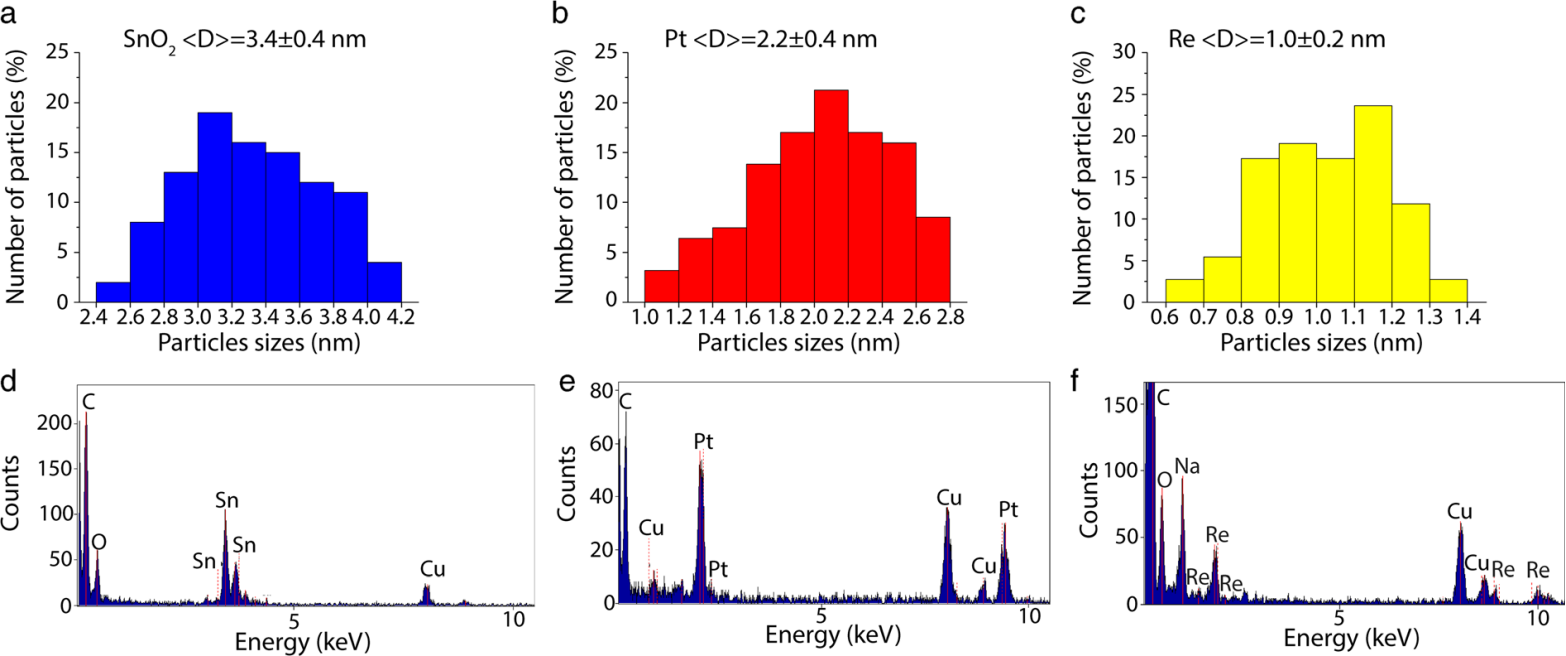
Upper row - particle size distribution calculated based on STEM images: **a** SnO_2_, **b** Pt, and **c** Re NPs; bottom row - EDS spectra of **d** SnO_2_, **e** Pt, and **f** Re NPs

The obtained SnO_2_, Pt, and Re nanoparticles were uniformly distributed on the respective TEM grids, which is confirmed by STEM overview images (Fig. 1a–c). The analyzed nanoparticles differ with size: the average diameters of the SnO_2_, Pt, and Re NPs are 3.35, 2.17, and 1.02 nm (Fig. 2a–c), respectively. The corresponding SAED patterns allowed identifying the crystalline structure of the analyzed nanoparticles. The electron-diffraction patterns were indexed using reference patterns for Re (PDF#870599), Pt (PDF#040802), and SnO_2_ (PDF#880287). The diffraction rings in the SAED patterns taken from the SnO_2_ and Pt NPs exhibit sharp rings containing some spots (Fig. 1d, e), while the Re diffraction pattern consists of very blurred rings (Fig. 1f). Thus, it can be concluded that SnO_2_ and Pt NPs are better crystallized than Re NPs. This is confirmed by the high-resolution HAADF STEM images (Fig. 1g–i). In the case of SnO_2_ and Pt NPs, well-defined lattice fringes, corresponding to SnO_2_ and Pt, are visible, while the Re atoms form small, not fully crystallized clusters. Some of them are spherical, but many of them have irregular shapes, consisting of five to six atomic planes reaching sizes below 1 nm. Moreover, many of the Re NPs are amorphous. The blurring of the diffraction pattern originates from the very small size of the NPs, yet the volume fraction of the amorphous Re NPs has also a contribution.

The SnO_2_ nanoparticles obtained by the three different synthesis methods were characterized in detail in (Drzymała et al. 2017); therefore, in this study, the best crystallized tin oxide NPs synthesized by the polyol method were chosen for further investigation.

The EDS spectra (Fig. 2d–f) confirm the presence of tin, platinum, and rhenium in the individually synthesized nanoparticles. The peaks from carbon and copper originate from the TEM grids. Additionally, this technique allowed to define the respective atomic percentages of the elements forming the binary (Pt/SnO_2_ and ReSnO_2_) and ternary (Pt/Re/SnO_2_) nanoparticle combinations. The atomic composition of the above-mentioned samples is compiled in Table 1.

**Table 1:** Atomic composition of binary and ternary combinations of nanoparticles determined by EDS

Sample	EDS (at.%)		
	Pt	Re	Sn
Pt/SnO_2_	80	–	20
Re/SnO_2_	–	20	80
Pt/Re/SnO_2_	55	10	35

### FTIR spectroscopy

**Fig. 3:**
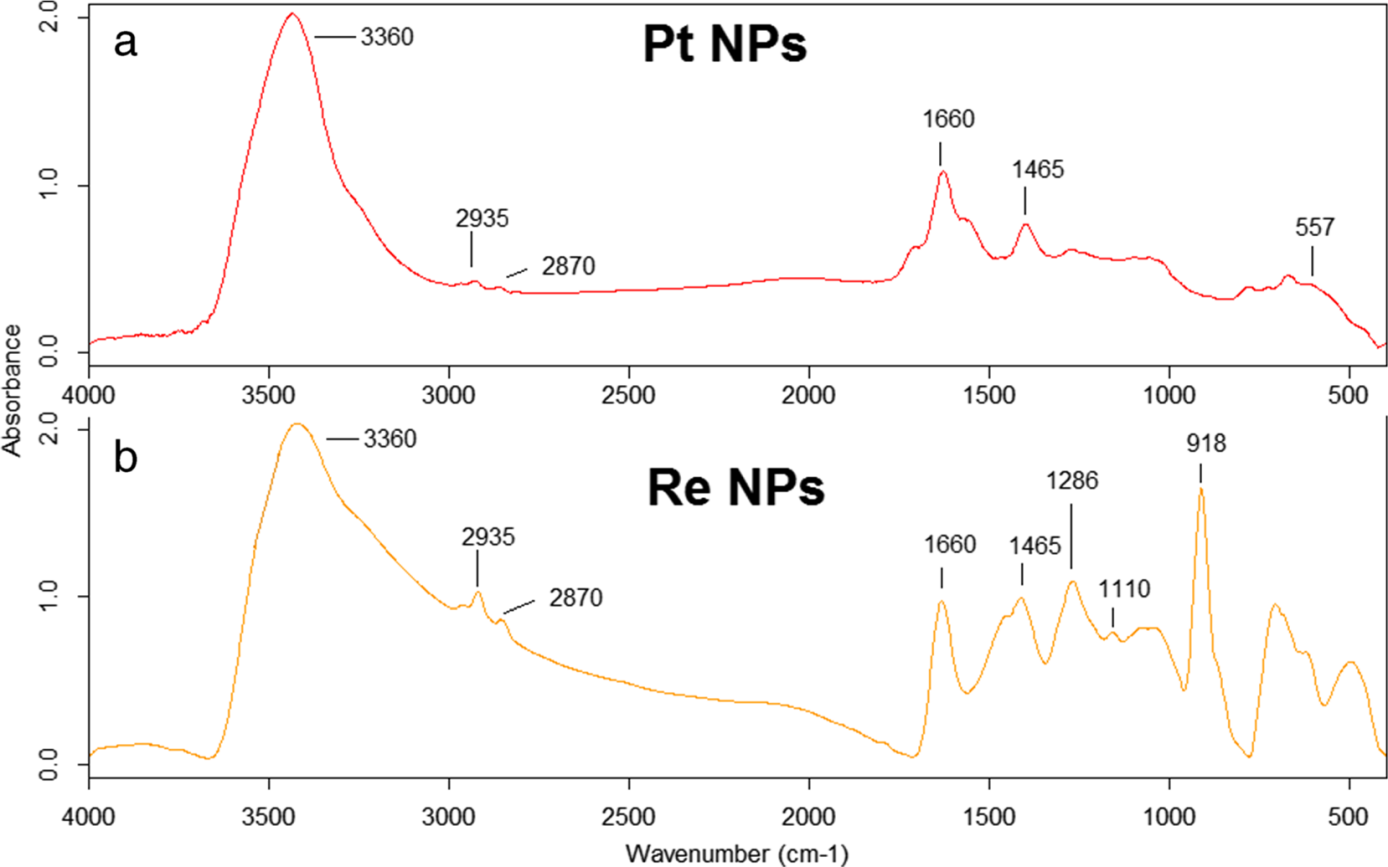
FTIR spectra of Pt NPs (**a**) and Re NPs (**b**)

Due to the fact that EDS can show only the presence or absence of elements such as Sn, O, Pt, and Re (Fig. 2d–f), FTIR spectroscopy as well as XPS (see supplementary information) were used to verify if the Pt and Re NPs are indeed metallic, as sometimes oxidation of Pt an Re occurs (Luo et al. 2004; Gong and Zhou 2009; Yang et al. 2016). A detailed FTIR analysis of SnO_2_ NPs was performed in Drzymała et al. (2017). Figure 3 shows the comparison of the FTIR spectra collected for Pt NPs (a) and Re NPs (b). In the FTIR spectra of Pt NPs (Fig. 3a), only absorption bands at 557 cm^−1^, corresponding to the stretching asymmetric vibrations of Pt–Cl, are present (Allen and Theophxnides 1964; Krishnakunar et al. 2008). XPS measurements confirmed the metallic character of the Pt NPs and showed also the presence of some Pt^2+^ species (Fig. S3).

In the FTIR spectrum of Re NPs, no band at 863.25 cm^−1^ corresponding to stretching vibrations of the Re–O bond was observed (Luo et al. 2004). However, several bands originating from organic compounds used for the synthesis and reduction of the Re NPs were found. Indeed, vibrations of outer face and inter face of OH group from PVP (wave numbers at 918 and 1286 cm^−1^, respectively) are visible (Sivaiah et al. 2010). Moreover, in the FTIR spectrum presented in Fig. 3b, a deformation vibration of the BH group from NaBH_4_ (1110 cm^−1^) is visible (Filinchuk and Hagemann 2008). In the FTIR spectra of the synthesized nanoparticles, vibrations from functional groups building ethylene glycol and ethanol are presented. The wave numbers at 1465 and 1660 cm^−1^ correspond to the bending vibrations of the CH_2_ group from ethylene glycol. Moreover, CH stretching symmetric and asymmetric vibrations (2870 and 2935 cm^−1^, respectively), in the FTIR spectra of Pt NPs (Fig. 3a), and of Re NPs (Fig. 3b) are visible. In these spectra, OH vibrations (3360 cm^−1^) from ethylene glycol and ethanol are noticed (Plyler 1952; Krishnan and Krishnan 1966). Moreover, these OH vibrations could originate from the protonation of Re NPs (Manbeck et al. 2015). All peaks presented in Fig. 3 are summarized in Table 2. XPS spectra confirmed the presence of metallic Re as well as minor amounts of Re oxides (see supplementary information).

**Table 2:** FTIR analysis of Pt NPs and Re NPs (Plyler 1952; Allen and Theophxnides 1964; Krishnan and Krishnan 1966; Luo et al. 2004; Krishnakunar et al. 2008; Gong and Zhou 2009; Yang et al. 2016)

Wave number (cm^−1^)	Bond/stretching functional group	Pt NPs	Re NPs
557	Pt-Cl vibrations		Not present
918	OH outer face vibration from PVP	Not present	
1110	BH deformation fromNaBH_4_	Not present	
1286	OH inter face bending vibration from PVP	Not present	
1465	CH_2_ bending from glycol		
1660	CH_2_ bending from glycol		
2870	CH stretching symmetric from glycol and PVP		
2935	CH stretching asymmetric from glycol and PVP		
3360	OH bending from glycol and ethanol or Re NP protonation		

**Fig. 4:**
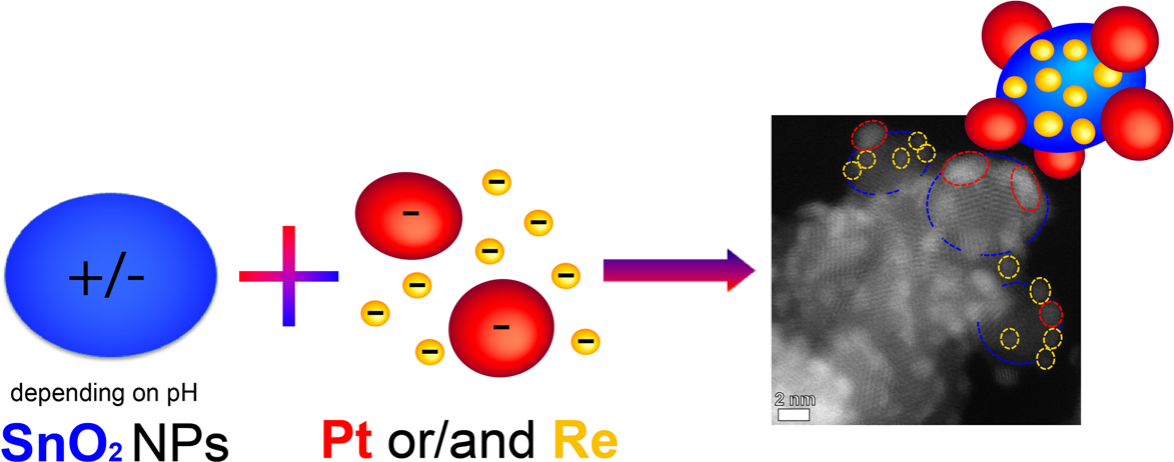
Schematic representation of the idea of controlled assembling of positively charged SnO_2_ NPs with negatively charged metallic Pt and Re NPs into ternary catalysts by adjusting their pH values

In order to assemble in a controlled way the individual NPs into binary and ternary combinations, their zeta potentials were measured. Subsequently, it was possible, by adjusting the pH value of the NPs containing solution, to obtain NPs with a desired positive or negative charge. In this way, positively charged NPs could be successfully assembled with negatively charged NPs to form binary and ternary combinations (Fig. 4).

### Zeta potential measurements

**Fig. 5:**
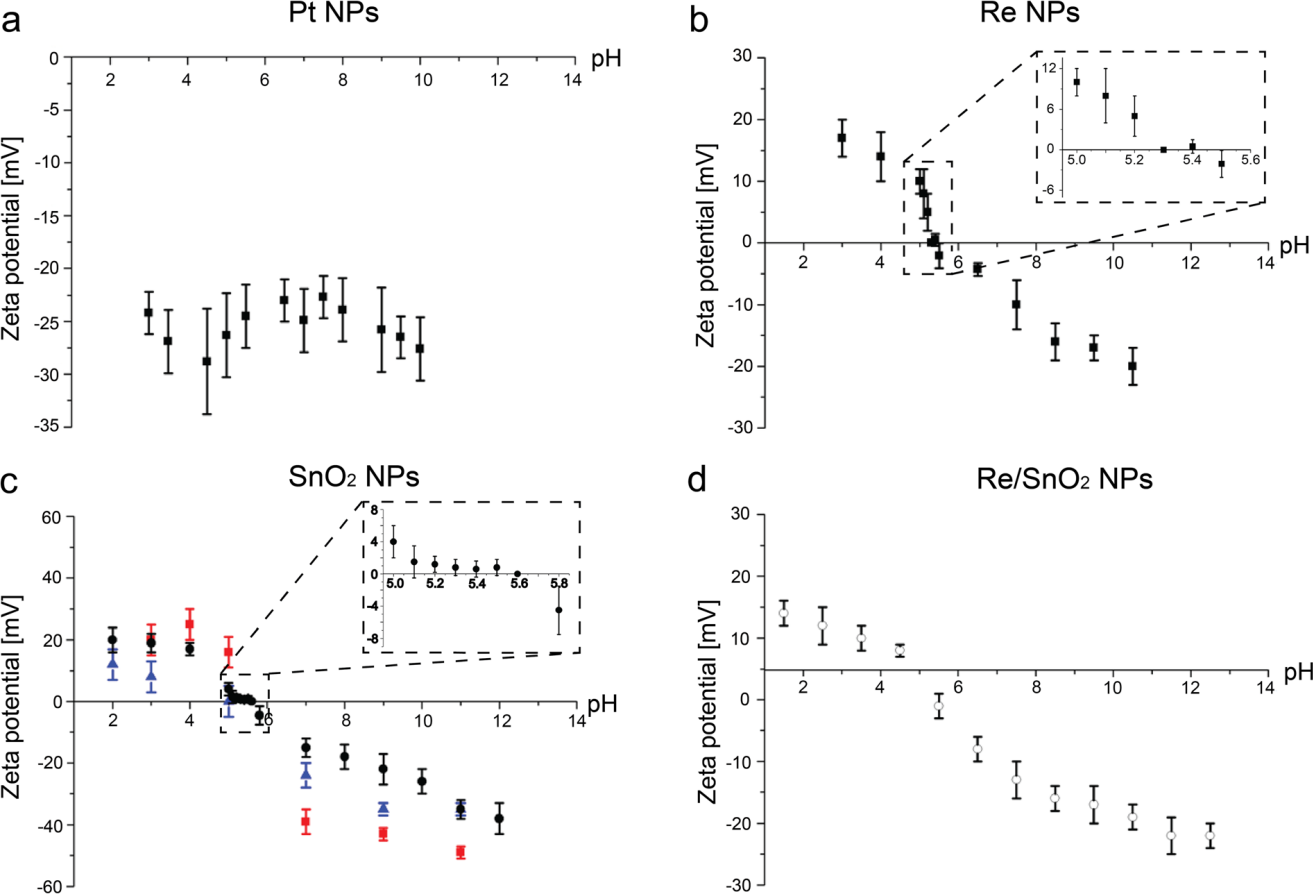
Dependence of zeta potential on the pH for three types of nanoparticles: **a** Pt NPs synthesized by polyol method; **b** Re NPs synthesized by the colloidal method; **c** SnO_2_ NPs synthesized by polyol method (circles), citric acid method (squares), and microwave-assisted method (triangles); and **d** for the Re/SnO_2_ combination. The inset plots show an enlargement of the data for the pH range 5–5.5

The zeta potential values of Pt, Re, and SnO_2_ dispersions as a function of their pH are presented in Fig. 5. The Pt NPs are negatively charged in the entire range of pH (Fig. 5a). Their zeta potential values range from about − 25 to − 30 mV and do not seem to depend on the pH value. The Re NPs are negatively charged at the initial pH = 5.3. Their zeta potential increases from about − 20 to + 17 mV with decreasing value of pH. At pH between 5.5 and 11, the Re NPs are negatively charged having zeta potentials starting at − 2.1 mV. The dependence of zeta potential of SnO_2_ NPs dispersions on their pH value (Fig. 5c) is similar for all applied synthesis routes. Their zeta potentials are positive for pH values below 5.5 and negative when the pH increases with the isoelectric point around pH = 5.6. For the critical range of pH between 5 and 5.8 containing the isoelectric point, more zeta potential data was collected and presented as insets in Fig. 5b, c.

As the zeta potential dependence on pH for all SnO_2_ nanoparticles seems to be independent on the synthesis method, in this study, SnO_2_ nanoparticles, which were synthesized by polyol method, were used for binary and ternary combinations, because they were the most crystalline, relatively large, and not agglomerated.

Additionally, the zeta potential of the binary Re/SnO_2_ combination as a function of the pH has been measured (Fig. 5d). The Re/SnO_2_ binary NPs are positively charged at pH values below 5. With increasing value of pH, their zeta potential decreases from about + 14 to − 22 mV passing the isoelectric point around pH = 5.5. The SnO_2_ nanoparticles, which have a larger size compared to Re NPs, may dominate the course of this curve. Therefore, the course is similar to the zeta potential of SnO_2_ nanoparticles synthesized using the polyol method.

### TEM morphology of binary combinations

**Fig. 6:**
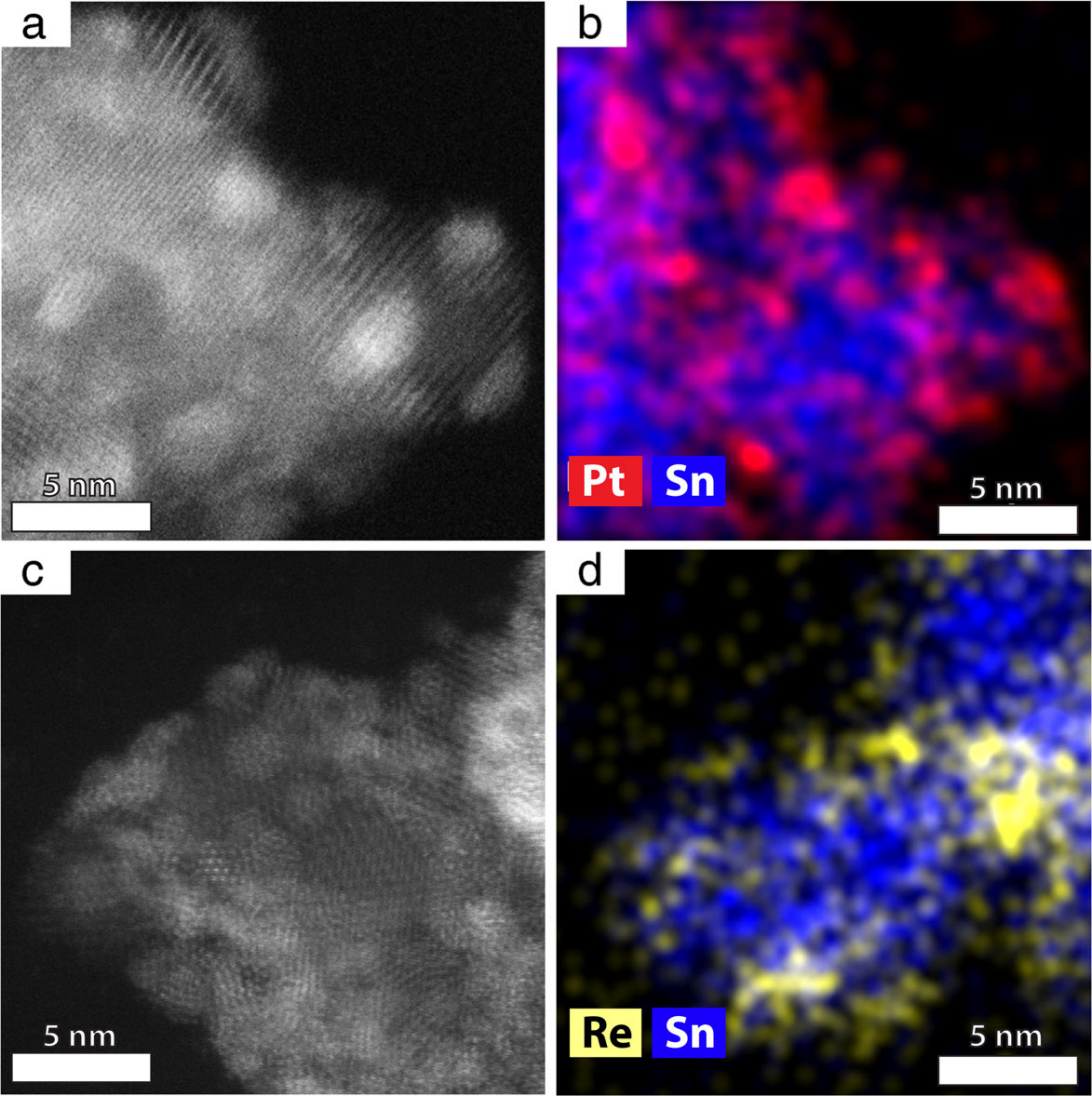
Binary combinations of Pt/SnO_2_ and Re/SnO_2_ nanoparticles: HR-HAADF STEM image of Pt/SnO_2_ (**a**), EDS map showing the chemical distribution of Pt and Sn (**b**), HAADF STEM image of Re/SnO_2_ (**c**), and EDS map showing the chemical distribution of Re and Sn (**d**). Please note that the EDS map is taken from a different location than the HRSTEM image in **c**

To produce binary Pt/SnO_2_ NPs, the pH of the respective solutions containing Pt NPs and SnO_2_ NPs had to be adjusted in order to have oppositely charged NPs. From the zeta potential measurements (Fig. 5), it was found that at pH value of 5, the Pt NPs had a negative charge of − 28 mV, conversely to SnO_2_ NPs, which were positively charged at + 4 mV. These opposite potential values assured a successful decoration of SnO_2_ NPs with Pt NPs. In order to obtain the binary combination of Re/SnO_2_ NPs (shown in Fig. 6c, d), the pH of the respective solutions containing Re NPs and SnO_2_ NPs were also adjusted. At pH = 5, the Re NPs had a positive charge of + 10 mV and SnO_2_ NPs were less positive with a charge of + 4 mV. These zeta potential values allowed for the attachment of Re NPs to SnO_2_ NPs, as confirmed by the TEM images (Fig. 6c, d). Thus, the Pt or Re NPs containing solutions were added drop-wise to the SnO_2_ NPs containing solution and sonicated and stirred overnight. The obtained Re/SnO_2_ mixture was used to assemble the three-component combination using the ternary assembly method described in the experimental part.

To verify if the binary NP combinations were successfully obtained, STEM HAADF images (Fig. 6a, c) were acquired from the mixed NPs containing solutions. Due to the fact that the contrast in the HAADF detector depends on *Z* number of the chemical elements constituting the sample, metallic, brighter nanoparticles are easily distinguished from SnO_2_ (darker nanoparticles). This observation is also completed by EDS maps, showing the distribution of Pt (Fig. 6b) or Re (Fig. 6d) on tin oxide NPs. Indeed, STEM analysis confirmed that after mixing together the solutions containing one type of NPs, either SnO_2_ nanoparticles decorated with platinum or rhenium nanoparticles were obtained, respectively.

**Fig. 7:**
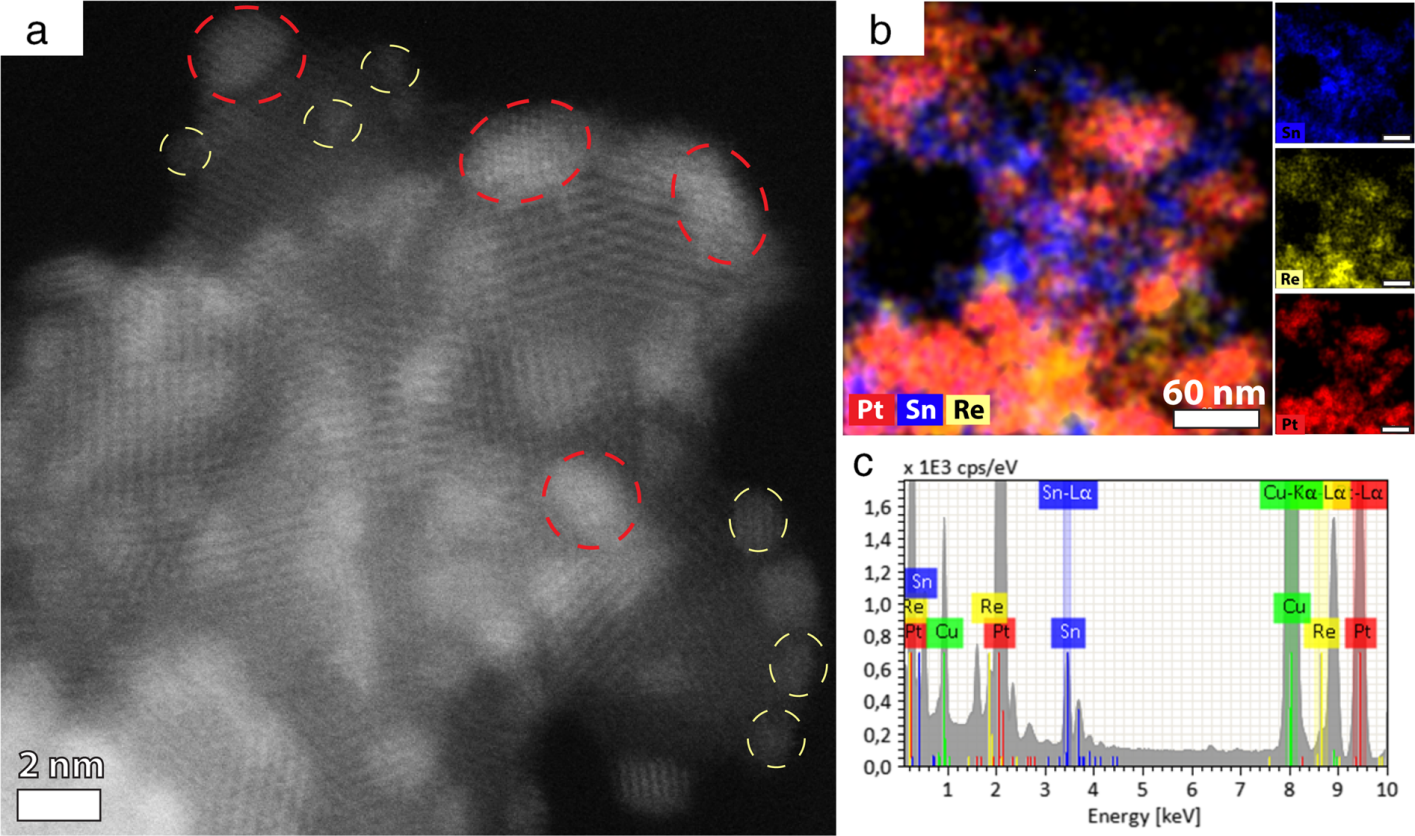
Pt/Re/SnO_2_ ternary combination of nanoparticles: HAADF HRSTEM image of Pt/Re/SnO_2_ (**a**). Platinum and rhenium nanoparticles were marked with red and yellow dashed circles, respectively. Elemental distribution of Pt, Re, and Sn (**b**). Please note that the EDS map is taken from a different location than the HRSTEM image. EDS spectrum (**c**)

The ternary PtRh/SnO_2_ sample was analyzed by HRSTEM HAADF (Fig. 7a). The sample consists of small Pt and Re NPs located on the surface of larger SnO_2_ NPs. Platinum and rhenium can be distinguished due to the brighter contrast and the smaller size of these nanoparticles compared to the tin oxide support. Both, the EDS elemental map and the EDS spectrum confirm the presence of all three components: Pt, Re, and Sn. The signal from copper in the EDS spectrum originates from the TEM grid. The EDS maps show that signals from Pt, Re, and Sn overlap; therefore, it is concluded that all nanoparticles were mixed together. In this way, the aimed ternary nanoparticle system containing Pt, Re, and SnO_2_ NPs was obtained.

## Discussion

In the preset study, Pt, Re, and SnO_2_ NPs were synthesized, and after structural characterization, their zeta potentials were measured as function of pH. To assemble the NPs into Pt/SnO_2_ and Re/SnO_2_ NPs, the NPs containing solutions were mixed together at a pH guaranteeing opposite zeta potentials of the metal and oxide NPs. STEM observations combined with EDS analysis confirmed the successful formation of binary and ternary NP combinations. In all cases, metallic nanoparticles were homogeneously distributed on tin oxide surfaces forming flower-like structures. These structures, in future, could provide access of ethanol molecules to active sites of ternary Pt/Re/SnO_2_ nanocatalyst, similarly to ternary PtRh/SnO_2_ nanocatalysts (Kowal et al. 2009a, b).

In IR range between 4000 and 400 cm^−1^, information about chemical bonds between the metal surface and the oxide is obtained. Therefore, to verify whether the synthesized nanoparticles are metallic or metal oxides, FTIR and XPS spectroscopies were used. In the FTIR spectra of synthesized Pt NPs and Re NPs, only vibrations from functional groups building chemical compounds used to reduce the precursors and washing the samples were visible. The vibrations of Pt–O and Re–O should be visible in the FTIR spectra at wave numbers 953 and 863 cm^−1^, respectively (Luo et al. 2004; Gong and Zhou 2009). In the FTIR spectra of the synthesized nanoparticles, these vibrations were not observed. Consequently, it can be stated that the analyzed nanoparticles were metallic. This information was also confirmed by XPS measurement (see supplementary information).

The determination of NP zeta potential is important to optimize the process of assembly of the aimed ternary nanoparticle system by electrostatic heteroaggregation. The charge of NP dispersion depends on its pH value, allowing connecting them into ternary nanostructures in a controlled way. The experimentally measured dependence of the zeta potentials on pH for the Pt, Re, and SnO_2_ nanoparticle dispersions is presented in Fig. 5. The zeta potential of the platinum nanoparticles is relatively high and negative in the entire range of the pH. Marzun et al. reported a pH of 1.7 as the isoelectric point of platinum nanoparticles stabilized by neutral polymer polyvinylpyrrolidone (MW = 58,000 g/mol) and the value of zeta potential between pH 3 and 12 not exceeding − 10 mV in the negative sense (Marzun et al. 2014). Lower values of zeta potentials (between − 25 and − 30 mV) observed in the present study can be attributed to use of ethylene glycol as stabilizer with lower molecular weight.

Data concerning zeta potential of rhenium nanoparticles is very limited in the literature. Pawlak et al. (2014) measured the zeta potential of rhenium sulfide micron-sized particles. They found negative zeta potential in the pH range between 3 and 9, which could be attributed to the sulfate groups at the interface. The negative charge of nanoparticles can originate from the ReO_4_^−^ surface groups (Kessler and Seisenbaeva 2012) and the use of PVP as a stabilizer leading to allow absolute value of the zeta potential. In the present study, the isoelectric point of Re NPs was observed at an approximate pH value of 5.5, which could suggest that the Re NPs might have OH groups adsorbed on their surface. Indeed, PVP used as a stabilizer provided these OH groups, which are visible in the FTIR spectra (Fig. 3b). In literature, it is often observed that Re complexes undergo protonation and deprotonation (Manbeck et al. 2015). At pH = 5, both Re NPs and SnO_2_ NPs have positive potentials (+ 10 and + 4 mV, respectively). STEM observations (Fig. 6c, d) confirm that although both NPs have positive zeta potentials, heteroaggregation to Re/SnO_2_ occurs. This aggregation happens due to the zeta potential difference between more positive Re clusters and weakly positive SnO_2_ NPs. Moreover, as the synthesized Re NPs are in form of ~1-nm clusters, therefore they have a high surface energy which consequently helps them to heteroaggregate on top of the SnO_2_ NPs. In the case of Pt NPs, which are larger (~2 nm) compared to Re NPs, indeed a smaller coverage of SnO_2_ NPs with Pt NPs is observed, although they exhibit opposite zeta potentials (Fig. 6a, b).

The isoelectric point of the SnO_2_ nanoparticles seemed not to be dependent on the synthesis method, and it was in the range given by Kosmulski (2009). At a pH value between 5 and 5.5, positively charged SnO_2_ NPs and negatively charged Re NPs could be observed. Therefore, mixing platinum NPs (negatively charged) with SnO_2_ and Re NPs at that pH caused their heteroaggregation. As a result of electrostatic interaction, multi-metal nanoparticle systems (Pt/Re/SnO_2_, Re/SnO_2_, and Pt/SnO_2_) were obtained.

## Conclusions

Pt, Re, and SnO_2_ NPs were individually synthesized by chemical methods leading to the formation of well-crystallized Pt (2 nm) and SnO_2_ (3.4 nm) NPs and of Re clusters of 1 nm. The aim was to interconnect them by heteroaggregation into binary and ternary combinations. For this purpose, the zeta potentials as a function of the pH of the individual NP solutions were measured. Except for Pt NPs, Re and SnO_2_ NPs exhibited a positive zeta potential at acidic pH with an isoelectric point between pH of 5 and 6 and becoming negative at basic pH. Only Pt NPs were negatively charged around − 24 mV in all the measured pH range. This could be due to ethylene glycol used as a stabilizer, which could be still present on the NP surface (Wu and Chen 2003). In order to successfully combine the NPs into Pt/SnO_2_ and Re/SnO_2_ NPs, the solutions were mixed together at a pH guaranteeing opposite zeta potentials. STEM observations combined with EDS confirmed the successful formation of binary NPs. By adding Pt into Re/SnO_2_, ternary Pt/Re/SnO_2_ NPs were formed. This study shows that by controlling the zeta potential of individual NPs, it is possible to assemble them into binary and ternary combinations.

## Electronic supplementary material

ESM 1 (PDF 606 kb)
