# Supplementary material for: Design and assembly of ternary Pt/Re/SnO2 NPs by controlling the zeta potential of individual Pt, Re, and SnO2 NPs
Source: J Nanopart Res. 2018 May 12;20(5):144. doi: 10.1007/s11051-018-4244-0 (PMC5949135; doi:10.1007/s11051-018-4244-0)
Supplement: Supplementary file 1 — (PDF 606 kb) [file 11051_2018_4244_MOESM1_ESM.pdf]

Supplementary information

Design and assembly of ternary Pt/Re/SnO_2 NPs by controlling the zeta potential of individual Pt, Re and SnO_2 NPs

Elżbieta Drzymała[1*], Grzegorz Gruzeł[1], Anna Pajor-Świerzy[2], Joanna Depciuch[1], Robert Socha[2], Andrzej Kowal[1], Piotr Warszyński[2] and Magdalena Parlinska-Wojtan[1]

[1]*Institute of Nuclear Physics Polish Academy of Sciences, PL-31342 Krakow, Poland*

[2]*Jerzy Haber Institute of Catalysis and Surface Chemistry Polish Academy of Sciences, Niezapominajek 8, PL-30239 Krakow, Poland*

[*]Corresponding author, e-mail: elzbieta.drzymala@ifj.edu.pl

Control experiment – Pt and Re NPs synthesized at other pH conditions

The synthesis of Pt was also performed at acidic pH (without adjusting it with NaOH), leading to nanoparticles having larger sizes with a non-uniform size distribution, which were more agglomerated (Fig. S1). The obtained nanoparticle suspension was not stable and the nanoparticles precipitated after a few hours. STEM observations of Re NPs synthesized at different pH values (Fig. S2) were performed. It turned out that during a synthesis conducted in an acidic environment, no nanoparticles are formed and only their jagged parts are present in the solution. On the other hand, in basic environment strongly agglomerated islands of nanoparticles on a TEM grid were obtained – therefore such highly agglomerated particles are not suitable for combining them with others. In the case of metallic nanoparticles (Re and Pt), TEM was sufficient to show that the synthesis carried out under other pH conditions leads to obtaining nanoparticles with non-uniform particle size distribution, larger sizes and often more agglomerated.

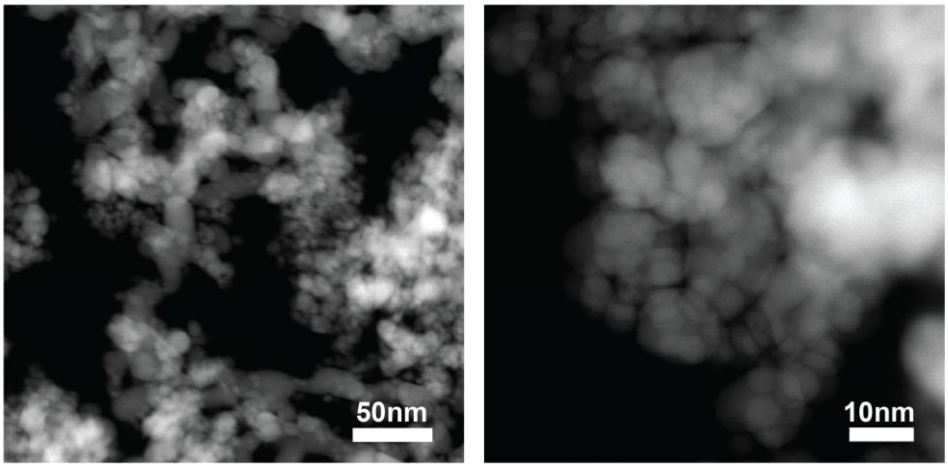

Fig. S1 Platinum nanoparticles synthesized in acidic pH.

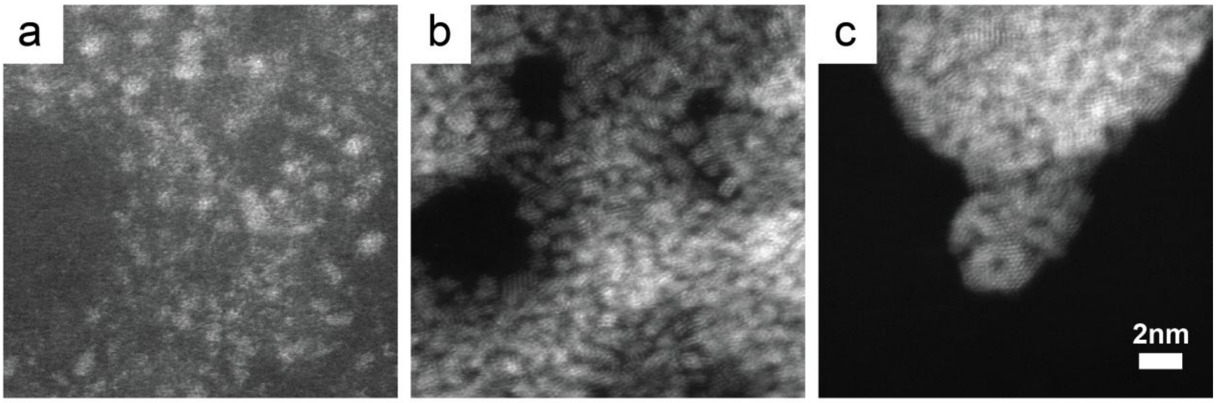

Fig. S2 Rhenium nanoparticles synthesized at various pH values: a) pH = 3, b) pH = 8, c) pH = 12. The scale bar corresponds to 2 nm in all pictures

XPS analysis

XPS analysis provided information about the potential catalyst surface composition. The oxidation states of Pt, Re, and Sn were studied by XPS following the Pt 4f, Re 4f, and Sn 3d transitions, respectively. Figure S3 shows the Sn 3d XPS spectra obtained for binary $ReSnO_2$ (a) and $PtSnO_2$ (b), as well as for the ternary $PtReSnO_2$ (c) combinations of NPs. The spectra are similar for all samples and display two main component peaks with respective binding energies of 487.25 eV and 495.5 eV. The Sn 3d spectra can be deconvoluted into three doublet peaks attributed to Sn-C at 494 and 485.3 eV, Sn (II/IV) at 495.7 and 487.3 eV and Sn^{4+}-Cl at 497.05 and 488.6 eV. The peaks at 495.7 and 487.3 eV originated from tetravalent tin proving the formation of SnO_2. Discriminating between Sn (II) and Sn (IV) oxides is difficult, because both species appear at very close binding energies. The Sn-C peak originates from the interface with the Vulcan XC-72R carbon support. The Sn^{4+}-Cl peak is related to the tetravalent tin surrounded by chlorine. Most probably bivalent tin was oxidized to tetravalent tin, however the final product, which is SnO_2 was not formed. Instead, tin was attached to chlorine. The electron states of tin on the surfaces of all analyzed samples are similar. The Re 4f spectrum profile is displayed in Fig. S3d. The XPS signal can be deconvoluted into three doublet peaks at BEs of 48.2 and 46.1 eV, 44 and 46.3 eV, 42.65 and 44.95 eV. The deconvoluted peaks centered at 42.65 and 44.95 eV are attributed to Re^0, although the BE is about 1.5 eV higher from the highest energy observed for metallic rhenium. It is related to the small size (~1 nm) of rhenium nanoparticles, confirmed by TEM images (Fig. 1 and Fig. 2); this is the so-called initial state effect. The other two components originate from Re^{7+} and Re^{4+}, respectively, which can be attributed to the presence of rhenium oxides (orange dashed line and blue dotted line in Fig S3d). The spectrum of Pt 4f contains doublets of $4f_{7/2}$ and $4f_{5/2}$ (Fig. S3e,f). Two paired peaks were obtained by the deconvolution of Pt 4f, which could be assigned to metallic Pt and Pt^{2+} species, respectively. Clearly, the Pt 4f in $PtSnO_2$/C had binding energies (BEs) of 74.70 and 71.40 eV for metallic Pt in $4f_{5/2}$ and $4f_{7/2}$ doublets, respectively. In case of $PtReSnO_2$/C the BEs were 74.60 and 71.30 eV for Pt^0 in $4f_{5/2}$ and $4f_{7/2.}$ The negative shifts in the BE could indicate the expansion of the Pt lattice parameter due to the incorporation of larger Sn atoms. Moreover, this could be related also to the presence of a peak from Pt^{2+}-O-Sn at BE=72.3 eV (red dashed line in Fig. S3e,f) (Drawdy et al. 1990).

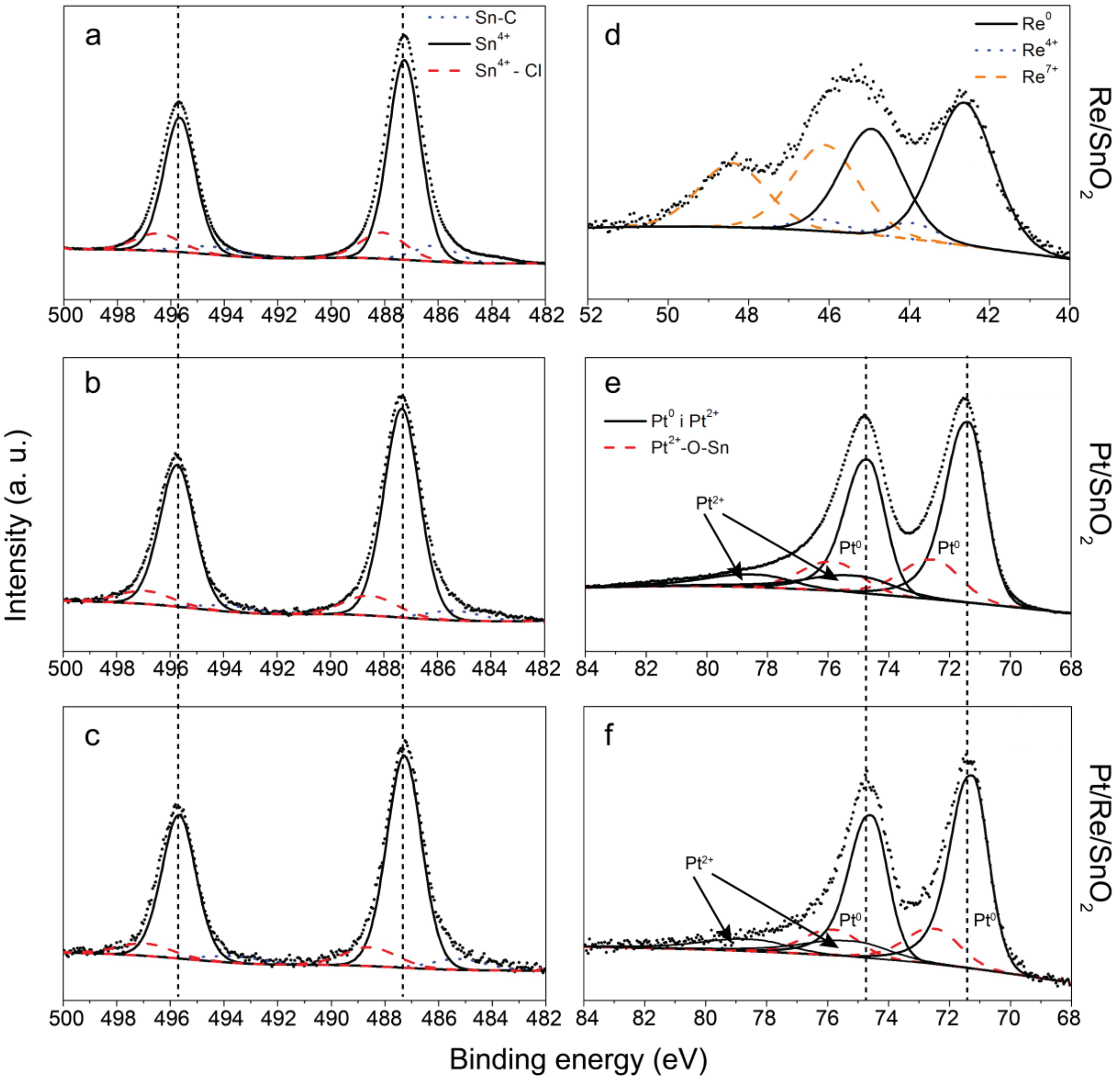

Fig. S3 XPS spectra of Sn 3d in (a-c) for $PtReSnO_2$, $PtSnO_2$ and $PtSO_2$, respectively, (d) Re 4f in $PtReSnO_2$ and $ReSnO_2$, Pt 4f in (e) $PtSnO_2$ binary and (f) $PtReSO_2$ ternary nanoparticle combinations.

References

[1] Drawdy JE, Hoflund GB, Gardner SD, Yngvadottir E, Schryer DR (1990) Effect of pretreatment on a platinized tin oxide catalyst used for low-temperature Co oxidation. Surf. Interface Anal. 16 (11-12): 369-374. doi: 10.1002/sia.740160178
